# Obstructive Sleep Apnea and Pulmonary Hypertension: A Chicken-and-Egg Relationship

**DOI:** 10.3390/jcm13102961

**Published:** 2024-05-17

**Authors:** Baran Balcan, Bahri Akdeniz, Yüksel Peker, The TURCOSACT Collaborators

**Affiliations:** 1 Department of Pulmonary Medicine, Koç University School of Medicine, Istanbul 34450, Turkey; mbalcan@kuh.ku.edu.tr; 2 Department of Cardiology, Dokuz Eylül University Faculty of Medicine, Izmir 35340, Turkey; bahri.akdeniz@deu.edu.tr; 3 Division of Pulmonary, Allergy, and Critical Care Medicine, University of Pittsburgh School of Medicine, Pittsburgh, PA 15213, USA; 4 Department of Clinical Sciences, Respiratory Medicine and Allergology, Faculty of Medicine, Lund University, 22185 Lund, Sweden; 5 Department of Molecular and Clinical Medicine, Institute of Medicine, Sahlgrenska Academy, University of Gothenburg, 40530 Gothenburg, Sweden

**Keywords:** obstructive sleep apnea, pulmonary hypertension, right-sided heart failure, positive airway pressure, narrative review

## Abstract

Obstructive sleep apnea (OSA) is characterized by repeated episodes of upper airway obstruction during sleep, and it is closely linked to several cardiovascular issues due to intermittent hypoxia, nocturnal hypoxemia, and disrupted sleep patterns. Pulmonary hypertension (PH), identified by elevated pulmonary arterial pressure, shares a complex interplay with OSA, contributing to cardiovascular complications and morbidity. The prevalence of OSA is alarmingly high, with studies indicating rates of 20–30% in males and 10–15% in females, escalating significantly with age and obesity. OSA’s impact on cardiovascular health is profound, particularly in exacerbating conditions like systemic hypertension and heart failure. The pivotal role of hypoxemia increases intrathoracic pressure, inflammation, and autonomic nervous system dysregulation in this interplay, which all contribute to PH’s pathogenesis. The prevalence of PH among OSA patients varies widely, with studies reporting rates from 15% to 80%, highlighting the variability in diagnostic criteria and methodologies. Conversely, OSA prevalence among PH patients also remains high, often exceeding 25%, stressing the need for careful screening and diagnosis. Treatment strategies like continuous positive airway pressure (CPAP) therapy show promise in mitigating PH progression in OSA patients. However, this review underscores the need for further research into long-term outcomes and the efficacy of these treatments. This review provides comprehensive insights into the epidemiology, pathophysiology, and treatment of the intricate interplay between OSA and PH, calling for integrated, personalized approaches in diagnosis and management. The future landscape of OSA and PH management hinges on continued research, technological advancements, and a holistic approach to improving patient outcomes.

## 1. Introduction

Sleep-related breathing disorders (SBDs) encompass a spectrum of conditions, each with distinct yet interrelated pathophysiological mechanisms. Among them, obstructive sleep apnea (OSA), central sleep apnea (CSA) with or without Cheyne–Stokes breathing, and sleep-related hypoventilation disorders are the most prominent [1]. Notably, OSA is recognized as a prevalent chronic condition deeply intertwined with various cardiovascular mechanisms [2]. The significance of these disorders extends beyond the realm of sleep medicine, emerging as critical components in the broader public health dialogue. This is especially true considering the escalating prevalence of obesity and aging populations worldwide, factors that are intrinsically linked with OSA [3]. The burgeoning evidence linking OSA with a myriad of cardiovascular complications underscores the necessity of a comprehensive understanding of this condition [4]. This review delves into the intricacies of OSA, focusing on its prevalence, impact on cardiovascular health, specific interplay with pulmonary hypertension, and the broader implications for patient management and treatment strategies.

### 1.1. Obstructive Sleep Apnea: A Chronic Condition with Significant Prevalence

Obstructive sleep apnea (OSA) is characterized by repeated episodes of partial or complete obstruction of the upper airway during sleep, leading to disrupted breathing patterns [1]. This disruption often results in significant, albeit transient, drops in blood oxygen levels—a condition known as nocturnal hypoxemia. The chronic nature of this hypoxemia in OSA patients is a major contributor to various cardiovascular complications [5]. Understanding the scale of this condition’s prevalence is essential for appreciating its public health implications. Epidemiological studies have shown that OSA affects a substantial proportion of the population, with prevalence rates varying based on demographic factors such as age, gender, and body mass index. The pervasiveness of OSA is further magnified when considering the often undiagnosed nature of the condition. Many individuals remain unaware of their condition, leading to a silent progression of associated comorbidities. Moreover, the evolving criteria for diagnosis and the advancements in screening methods are continuously reshaping our understanding of OSA’s true prevalence. This section aims to dissect the prevalence data, highlighting the epidemiological trends and the factors contributing to the widespread nature of OSA.

The prevalence of OSA is alarmingly high, impacting diverse demographic groups. Studies indicate varying rates, with a general consensus that it affects approximately 20–30% of males and 10–15% of females in the general adult population [6]. However, these numbers increase significantly with age and obesity. For instance, in populations over the age of 60, the prevalence of OSA can be as high as 60–70%. This is particularly concerning given the aging global population and the increasing rates of obesity worldwide [6]. Moreover, the relationship between OSA and body mass index (BMI) is well established. Obesity, particularly central obesity, is a major risk factor for OSA [7]. The distribution of body fat around the neck and upper airway contributes to airway obstruction during sleep. This correlation highlights the need for integrated public health strategies that address both obesity and OSA as interconnected health issues. Gender differences in OSA prevalence are also notable [3,8]. Traditionally, OSA has been viewed as a predominantly male disorder. However, recent studies suggest that this gender disparity may be partly due to differences in symptom presentation and diagnostic criteria [3]. Women often present with atypical symptoms, leading to underdiagnosis. As the awareness and understanding of these gender-specific presentations increase, it is likely that the reported prevalence in women will rise. Additionally, the impact of genetic factors on OSA prevalence cannot be overlooked [9]. Familial studies suggest a genetic predisposition to OSA, with certain anatomical and functional traits being heritable. This adds another layer of complexity to understanding OSA’s prevalence and underscores the necessity for personalized approaches in both diagnosis and treatment [2,10].

### 1.2. Impact on Cardiovascular Health

The cardiovascular implications of OSA are far-reaching and complex. This includes systemic hypertension, coronary artery disease, heart failure, and arrhythmias. The mechanisms underlying these cardiovascular sequelae are multifactorial, involving sympathetic nervous system activation, oxidative stress, systemic inflammation, and endothelial dysfunction [11]. The connection between OSA and systemic hypertension is particularly well documented [12]. Repeated episodes of nocturnal hypoxemia and hypercapnia lead to increased sympathetic nervous system activity, resulting in elevated blood pressure. This sympathetic surge not only occurs during sleep but can persist into the daytime, contributing to sustained hypertension. Coronary artery disease and heart failure are also notable consequences of OSA [13]. The chronic state of low oxygenation and the resultant stress on the cardiovascular system can exacerbate or even initiate the development of these conditions. Furthermore, the recurrent arousals from sleep, a hallmark of OSA, contribute to an overall state of increased stress and inflammation, factors known to be detrimental to cardiovascular health. Arrhythmias, particularly atrial fibrillation, have a strong association with OSA. The fluctuations in intrathoracic pressure, coupled with the autonomic imbalances caused by OSA, create an arrhythmogenic environment within the heart. This is especially critical in patients with existing cardiac conditions, where OSA can significantly increase the risk of arrhythmic events.

### 1.3. Pulmonary Hypertension: Definition and Classification

Pulmonary hypertension (PH) is a serious condition characterized by elevated pressure in the pulmonary circulation. Clinically, it is defined as a mean pulmonary artery pressure (mPAP) exceeding 20 mmHg at rest, as measured by right heart catheterization [14]. However, echocardiography is also effective in the diagnosis of PH and differentiating PH from other cardiac conditions [15]. This condition can lead to significant morbidity and mortality if left untreated. PH is categorized into five groups based on its etiology and pathophysiology. Pulmonary arterial hypertension (PH), a subset of PH, is particularly noteworthy due to its idiopathic nature and its association with various systemic diseases [16]. The pathophysiology of PH involves a complex interplay of various factors leading to increased pulmonary vascular resistance. This resistance is primarily due to pathological changes in the pulmonary arterioles, including vasoconstriction, thrombosis, and vascular remodeling. These changes result in right ventricular (RV) dilatation, systolic dysfunction, and ultimately, RV failure [17]. The classification of PH is crucial for guiding treatment decisions. Group 1 includes PH, which is idiopathic or linked to connective tissue diseases, congenital heart diseases, and other conditions. Group 2 encompasses PH due to left heart disease, while Group 3 includes PH due to lung diseases and/or hypoxia, under which OSA falls. Group 4 is chronic thromboembolic pulmonary hypertension (CTEPH), and Group 5 includes PH with unclear or multifactorial mechanisms [14]. Understanding this classification is essential for clinicians to appropriately diagnose and manage PH, particularly in the context of OSA, where the two conditions can have significant interplay.

### 1.4. Obstructive Sleep Apnea and Pulmonary Hypertension: An Intricate Relationship

Historically, OSA was categorized under Group 3 PH, signifying a direct relationship between these two conditions. This relationship is complex, with OSA contributing to the development and progression of PH. The frequent arousals and disrupted sleep patterns lead to sympathetic nervous system activation, which can further increase pulmonary artery pressures. The potential for therapeutic interventions in OSA to mitigate the effects on PH is a topic of significant clinical interest. Treatments such as continuous positive airway pressure (CPAP) have been shown to have beneficial effects on the pulmonary vasculature, potentially reducing the severity of PH in patients with OSA [18,19].

Understanding the pathophysiological mechanisms underlying the relationship between obstructive sleep apnea (OSA) and pulmonary hypertension (PH) is pivotal for comprehending their clinical interplay. This section delves into the complex physiological alterations initiated by OSA that pave the way for the development of PH.

## 2. Pathophysiology

### 2.1. The Interplay between Obstructive Sleep Apnea Mechanisms and Pulmonary Hypertension Development

**Figure 1:**
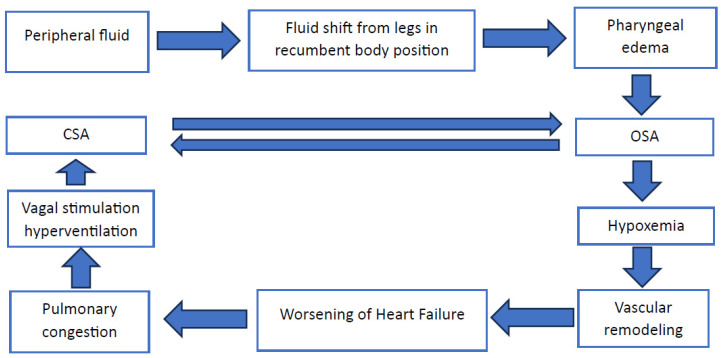
Interplay of obstructive sleep apnea and pulmonary hypertension. Abbreviations: CSA = central sleep apnea; OSA = obstructive sleep apnea.

OSA is marked by repeated episodes of significant upper airway obstruction during sleep. These obstructions, often lasting more than ten seconds, create a unique and harsh environment within the thoracic cavity, characterized by profound negative pleural pressures, sometimes reaching as low as −60 mmHg [20]. This physical strain has far-reaching physiological consequences, extending beyond mere sleep disturbance (Figure 1). The intermittent hypoxia experienced by OSA patients during sleep is not a benign occurrence; it sets off a cascade of pathophysiological responses that can culminate in significant cardiovascular morbidity [21]. Given the risk for pharyngeal edema due to the fluid shift from legs in recumbent body position [20], it is possible that PH may worsen due to the worsening of OSA severity in the supine position.

#### 2.1.1. Hypoxemia and Hypercarbia: Triggering Hypoxic Pulmonary Vasoconstriction

**Figure 2:**
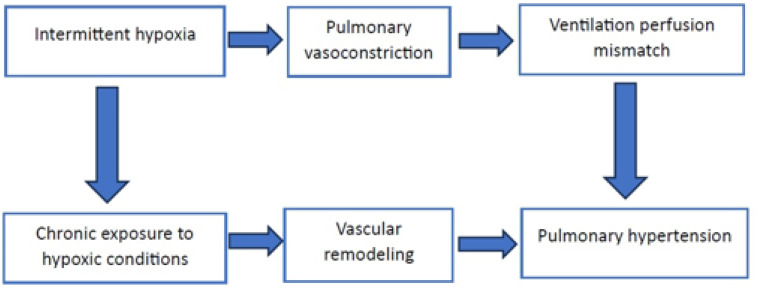
Mechanism of pulmonary vascular remodeling and pulmonary hypertension.

The hallmark physiological alteration in OSA is intermittent hypoxia—the cyclical drop and subsequent restoration of oxygen levels. Hypoxic pulmonary vasoconstriction, a reflex response of the pulmonary vasculature to low oxygen levels, leads to constriction of blood vessels in the lungs [22]. This mechanism, initially protective in nature, becomes detrimental in the chronic setting of OSA. However, in the setting of OSA, this response becomes maladaptive, contributing to increased pulmonary artery pressure and vascular remodeling. Sleep fragmentation in OSA also exacerbates PH. Over time, the persistent vasoconstriction exacerbates the mismatch between ventilation and perfusion, contributing significantly to the development of PH (Figure 2). This mechanism is particularly detrimental in patients with existing cardiovascular disease, where OSA can worsen the clinical course of PH.

#### 2.1.2. Increased Intrathoracic Pressure and Arousals

The increased intrathoracic pressure induced by OSA significantly impacts cardiac function. It affects heart rate variability and can lead to fluctuations in cardiac output. Moreover, the sleep fragmentation caused by frequent arousals during the night contributes to sympathetic nervous system activation, further influencing cardiovascular health [23]. This activation leads to a series of events, including increased heart rate, blood pressure, and vascular tone, thereby exacerbating the pulmonary hypertension.

### 2.2. Pulmonary Vascular Remodeling in Hypoxic Conditions

Chronic exposure to hypoxic conditions, as seen in OSA, prompts a significant response in the pulmonary vasculature [24]. Vascular remodeling in this context refers to structural changes within the pulmonary arteries’ walls, leading to an increased resistance to blood flow and elevated pulmonary arterial pressure. This remodeling process involves the proliferation of smooth muscle cells and fibroblasts, thickening of the arterial walls, and narrowing of the vessel lumen (Figure 2).

#### 2.2.1. Cellular Mechanisms: Proliferation vs. Apoptosis

Underlying the process of vascular remodeling is a disruption in the balance between cellular proliferation and apoptosis [14]. Chronic hypoxia tends to favor proliferation, resulting in the thickening of the vascular wall and narrowing of the vessel lumen. This remodeling not only increases resistance within the pulmonary vasculature but also sets off a cycle where increased pressure promotes further remodeling, exacerbating the condition [25]. Hypoxia-induced differentially expressed in chondrocytes (DEC1) inhibits peroxisome proliferative activated receptor-γ (PPARγ), a recognized protective factor of PH, and that this is a predominant mechanism underpinning oxidative stress and inflammatory responses in pulmonary arterial smooth muscle cells (PASMCs) during PH [26].

#### 2.2.2. Mediators of Remodeling: Imbalance in Vasoactive Substances

The remodeling process is mediated by a complex interplay of various vasoactive substances. Increased levels of endothelin 1, angiotensin II, and thromboxane A2 promote vasoconstriction and increased vascular tone. In contrast, the levels of nitric oxide (NO) and prostaglandins, which usually act as vasodilators, decrease in concentration under hypoxic conditions. This imbalance plays a crucial role in the pathogenesis of PH in OSA, leading to a perpetuated cycle of vascular changes and increased pulmonary pressures [27].

### 2.3. Autonomic Nervous System Dysregulation in Obstructive Sleep Apnea and Pulmonary Hypertension

OSA’s impact on the autonomic nervous system is a critical element in the pathophysiology of PH [28]. The recurrent apneic episodes lead to sympathetic overactivity parasympathetic dysfunction. Chronic intermittent hypoxia in OSA results in sustained sympathetic nervous system overactivity. This sympathetic overdrive contributes to systemic and pulmonary arterial hypertension by increasing vascular tone and heart rate. Disruption in the balance between sympathetic and parasympathetic activity can also occur, leading to cardiovascular instability and contributing to the pathogenesis of PH.

### 2.4. Role of Inflammatory Pathways

Inflammation plays a significant role in the pathophysiology of both OSA and PH. OSA is associated with elevated levels of systemic inflammatory markers like tumor necrosis factor-alpha (TNF-α) and interleukins [27,28]. These inflammatory cytokines contribute to endothelial dysfunction and may play a role in the vascular remodeling process seen in PH. In addition to systemic inflammation, local pulmonary inflammatory responses are also evident in OSA, further contributing to the pathophysiology of PH. This local inflammation can exacerbate pulmonary vascular remodeling and increase pulmonary vascular resistance.

### 2.5. Impact of Oxidative Stress

The role of oxidative stress, exacerbated by intermittent hypoxia in OSA, extends beyond direct vascular effects. Oxidative stress can directly injure pulmonary vascular endothelial cells, leading to dysfunction and contributing to vascular remodeling and PH. Oxidative stress interacts with inflammatory pathways, amplifying the inflammatory response and contributing to the progression of PH [24].

### 2.6. Metabolic Disturbances in Obstructive Sleep Apnea and Pulmonary Hypertension

OSA is often associated with metabolic disturbances that may contribute to the development of PH [29]. OSA is linked with insulin resistance and a higher prevalence of type 2 diabetes, both of which can contribute to cardiovascular disease and PH. Abnormalities in lipid metabolism seen in OSA patients may also contribute to vascular inflammation and endothelial dysfunction, further exacerbating PH.

### 2.7. Role of Right Ventricular Function

The impact of OSA on right ventricular function is an emerging area of interest in understanding the development of PH. The increased pulmonary pressures in PH place strain on the right ventricle, which can lead to right ventricular hypertrophy and dysfunction. Right ventricular dysfunction can lead to a decrease in cardiac output, exacerbating the hypoxic and hypercapnic states seen in OSA and creating a vicious cycle contributing to the severity of both OSA and PH.

## 3. Prevalence

### 3.1. Prevalence of Pulmonary Hypertension among Patients with Obstructive Sleep Apnea

The prevalence of PH in patients with OSA is a subject of significant clinical interest, as it has implications for both diagnosis and management. Studies have shown a wide range of prevalence rates, influenced by factors such as the severity of OSA, methods of measuring pulmonary artery pressure (PaP), and the presence of comorbidities [30].

#### 3.1.1. Variability in Prevalence Rates

Early research indicated transient and sometimes severe elevations in PaP during sleep in patients with OSA. However, whether these episodic increases in pressure lead to persistent daytime PH and subsequent right heart failure remains a topic of ongoing investigation. The reported prevalence rates of PH in OSA patients vary dramatically, from as low as 15% to as high as 80% [31,32]. This variance is attributed to differences in study designs, diagnostic criteria, and the patient populations examined. Such diversity underscores the complex nature of the relationship between OSA and PH, highlighting the need for a nuanced understanding of how these conditions intersect [33].

#### 3.1.2. Diagnostic Challenges

The assessment of PaP in OSA patients is typically performed using Doppler Echocardiography, which is less invasive but also less accurate compared to right heart catheterization (RHC), the gold standard. Many studies rely on echocardiography, which may not provide the most precise measurement of PaP, leading to the potential underestimation or overestimation of PH prevalence [33]. This diagnostic complexity necessitates a cautious interpretation of prevalence data and calls for more rigorous and standardized diagnostic approaches.

#### 3.1.3. Case Studies and Findings

Several notable studies have contributed to our understanding of PH prevalence in OSA patients. Only a handful of studies employed RHC [17,22]. The reported prevalence of PH can be skewed, especially if the data mostly come from patients undergoing cardiac catheterization. Minai et al., who researched 83 OSA patients undergoing RHC soon after diagnosis, found that 70% had PH (mean PAP > 25 mmHg), 22% had precapillary PH (PCWP < 15 mmHg), and 10% had severe PH (mPAP > 40 mmHg) [30]. In another study involving 100 participants, Laks et al. determined that PH is prevalent in moderate-to-severe OSA patients with coexisting obstructive lung disease [34]. Another larger study examining 220 OSA patients via RHC found that only 17% had PH, lightly linking OSA severity with PH. Here, PH was most commonly associated with obstructive lung disease, hypoxemia, and hypercapnia [35]. Sanner et al., studying 92 OSA patients who all underwent RHC, highlighted that OSA might independently heighten the risk for mild PH [36]. They also observed that in those with normal left ventricular pressure, PH correlates with OSA severity. Shared risk factors for PH and OSA include obesity and aging, complicating the assessment of direct causality. Concurrent chronic pulmonary disease, left ventricular diastolic dysfunction, and left atrial enlargement can also manifest in both conditions. Bady et al., in a study with 44 participants, reported that PH prevalence in OSA patients was correlated with obesity severity and related respiratory mechanical effects [2].

### 3.2. Prevalence of Obstructive Sleep Apnea among Patients with Pulmonary Hypertension

Conversely, evaluating the prevalence of OSA in patients with PH offers additional insights into their interrelationship. The prevalence of sleep-disordered breathing, including OSA, appears to be significantly higher in the PH population compared to the general population [30,34].

#### 3.2.1. Obstructive Sleep Apnea Prevalence in Pulmonary Hypertension Patients

One trial highlighted a stark difference in OSA prevalence between precapillary PH patients (68%) and controls (5%) [37]. A more recent single-center observational study reported a 25% prevalence of OSA in PH patients [38]. Even though nocturnal hypoxemia is commonly observed and ties to structural RV remodeling in PH, OSA severity is not necessarily linked to nighttime SpO2, clinical, or functional status. This might explain recent findings that nocturnal hypoxemia, but not sleep apnea, portends a worse prognosis [39] (Table 1).

#### 3.2.2. Screening and Diagnostic Recommendations

While comprehensive screening of all OSA patients for PH is not universally endorsed, the American College of Chest Physicians (ACCP) and ESC/ERS guidelines suggest that patients diagnosed with pulmonary arterial hypertension be screened for sleep-disordered breathing. If sleep-disordered breathing or hypoventilation is suspected, further tests, such as polysomnography or overnight oximetry, are advised [31]. The 2022 ESC/ERS guidelines now recommend referencing hypoventilation syndromes over SDB in Group 3, as conditions heightening the risk for PH [14]. Isolated nocturnal OSA typically does not cause PH, but those with hypoventilation syndromes leading to daytime hypercapnia often experience it.

## 4. Prognosis

The prognosis of patients with OSA who develop PH is a multifaceted issue, influenced by a range of factors, including the severity of each condition, comorbidities, and treatment efficacy.

### 4.1. Impact of OSA on Pulmonary Hypertension Prognosis

In patients with OSA, the development of PH is generally associated with mild-to-moderate increases in pulmonary artery pressure (PaP). However, certain demographic and clinical factors, such as gender, age, obesity, and the presence of nocturnal hypoxia, have been identified as correlating with a higher risk of PH [14]. The interplay of these factors can significantly influence the prognosis, often complicating the clinical course and management strategies.

### 4.2. Severity and Comorbidities

Severe PH is relatively rare in OSA patients, typically occurring only in the presence of other comorbid conditions like chronic obstructive pulmonary disease (COPD) or obesity. The presence of these comorbidities not only exacerbates the severity of PH but also complicates its management and worsens the overall prognosis. The interaction between OSA, PH, and comorbidities like COPD creates a challenging clinical scenario, necessitating a comprehensive and multidisciplinary approach to patient care.

### 4.3. Right Ventricular Failure

The progression to right ventricular failure in OSA patients with PH is uncommon but possible, especially in the presence of left-sided heart disease or chronic hypoxic respiratory disease. The chronic pressure overload on the right ventricle due to elevated pulmonary pressures can lead to right ventricular hypertrophy and, eventually, failure [40]. This progression underscores the importance of early detection and management of PH in patients with OSA to prevent these severe cardiac complications. The relationship between OSA severity and right ventricular (RV) function is elucidated through the measurement of myocardial performance index (MPI), a numeric value a key indicator of cardiac function defined as the sum of isovolumetric contraction time (ICT) and isovolumetric relaxation time (IRT) divided by ejection time (ET) and could be calculated for each ventricle individually [41]. Notably, RV MPI was statistically higher in moderate-severe OSA patients (0.62 ± 0.18) compared to mild OSA patients (0.50 ± 0.10) and controls (0.48 ± 0.08, *p* ≤ 0.001), highlighting the detrimental impact of OSA severity on RV function [42]. This is further evidenced by the presence of right ventricular diastolic dysfunction exclusively in moderate-severe OSA patients, underscoring the specific cardiovascular risks posed by more severe forms of OSA. Moreover, sleep apnea was independently associated with a depressed right ventricular ejection fraction, as determined by radionuclide ventriculography, even after adjusting for critical factors such as lung function, age, body mass index, sex, blood gas analysis, pulmonary artery pressure, and left ventricular ejection fraction. This association emphasizes the independent role of sleep apnea in compromising RV function, beyond the influences of demographic and physiological variables [36].

### 4.4. Prognostic Indicators

Studies have shown that OSA patients with pulmonary hypertension exhibit certain clinical features that can serve as prognostic indicators [43,44]. For instance, a shorter distance in the 6-min walk test and reduced survival rates over time have been observed in these patients compared to OSA patients without PH. These indicators can be crucial in assessing the severity of the disease and planning appropriate management strategies. Notwithstanding, there are no identified specific biomarkers with a prognostic role in this category of patients.

### 4.5. Gender, Age, and Obesity

The correlation of gender, younger age, and obesity with the development of PH in OSA patients is noteworthy [45,46,47]. This demographic trend suggests that these groups may be at a higher risk, necessitating more vigilant screening and potentially aggressive therapeutic approaches. Understanding these demographic correlations is essential for developing targeted interventions and preventative measures.

## 5. Impact of Obstructive Sleep Apnea Treatment on Pulmonary Hypertension

The treatment of OSA has significant implications for the management of PH. Various therapeutic strategies, particularly CPAP, have been evaluated for their impact on pulmonary hemodynamics in OSA patients.

### 5.1. Nocturnal Oxygen Therapy and Hypoxemia Correction

One of the primary treatments for OSA involves correcting hypoxemia, particularly through nocturnal oxygen therapy [48]. This approach aims to alleviate the intermittent hypoxia characteristic of OSA, thereby reducing the hypoxic pulmonary vasoconstriction that contributes to PH. The administration of nocturnal oxygen has been shown to improve oxygen saturation during sleep, which can potentially attenuate the progression of PH. However, the overall effectiveness of oxygen therapy in reversing established PH is still a subject of ongoing research.

### 5.2. Efficacy of Oxygen Therapy

The efficacy of oxygen therapy in the context of OSA and PH is a complex issue. While it undoubtedly improves nocturnal oxygenation, the long-term effects on pulmonary artery pressure and right heart function are less clear. Some studies have shown a reduction in pulmonary artery pressures with long-term oxygen therapy, while others have found no significant change [49]. This variability highlights the need for individualized treatment plans and further research into the optimal use of oxygen therapy in this patient population.

### 5.3. Continuous Positive Airway Pressure Treatment

CPAP, the standard treatment for OSA, works by providing a continuous flow of air through a mask to keep the airways open during sleep. This treatment not only improves sleep quality and reduces daytime sleepiness but also has potential implications for pulmonary hypertension. By preventing airway collapse and subsequent hypoxemic episodes, CPAP might reduce the pulmonary vascular reactivity to hypoxia and thereby mitigate PH [50] (Table 1). It is also crucial to accurately determine the precise CPAP pressure, particularly through a second-night titration, to establish the exact pressure required to effectively treat the patient’s OSA.

### 5.4. Effect on Pulmonary Arterial Pressure

Several studies have explored the effect of CPAP on pulmonary arterial pressure (PaP) in OSA patients. The rationale is that by preventing airway collapse and subsequent hypoxemic episodes, CPAP might reduce the pulmonary vascular reactivity to hypoxia and thereby mitigate PH [51]. The results from these studies have been mixed. In some cases, there was a non-significant decrease in the mean PaP after long-term CPAP use, while other studies reported notable reductions in pulmonary vascular resistance [52] (Table 1).

### 5.5. Prospective and Randomized Studies

Prospective studies utilizing RHC have shown mixed results regarding the impact of CPAP on PH in OSA patients. Some studies have observed a significant decrease in pulmonary artery pressure after several months of CPAP treatment, while others have reported minimal changes [52]. These mixed results suggest that while CPAP may benefit some patients with OSA and PH, it may not be universally effective in reducing pulmonary artery pressures.

### 5.6. CPAP and Long-Term Outcomes

The long-term impact of CPAP on the progression of PH and right ventricular function in OSA patients remains an area of active research. While short-term studies have demonstrated potential benefits, the long-term implications are less clear [40]. Further research is needed to understand the extent to which CPAP therapy can alter the long-term prognosis of PH in OSA patients.

### 5.7. Echoing the Results with Echocardiography

Case-controlled studies using echocardiography have reported a meaningful decline in PaP following consistent CPAP treatment [18,19]. These findings, although promising, need to be interpreted with caution due to the limitations of echocardiography in accurately measuring PaP.

### 5.8. Other Treatment Modalities of OSA

There is a lack of data regarding the effect of surgery or mandibular advancement device (MAD) for OSA on the prognosis of concomitant PH.

## 6. Discussion—Summary of the Key Findings and Future Research Directions

Pathophysiology: The intermittent hypoxia characteristic of OSA triggers a cascade of molecular and cellular events leading to oxidative stress, endothelial dysfunction, and vascular remodeling—all contributing to the development and progression of PH.

Prevalence: The prevalence rates of PH among OSA patients and vice versa underscore the significance of this comorbidity. Variability in these rates reflects the challenges in diagnosis and the influence of factors like age, obesity, and comorbid conditions. There is a lack of data regarding the prevalence of PH in OSA phenotypes such as stage-dependent and position-dependent OSA.

Prognosis: Prognostic indicators in OSA-related PH, including demographic factors and severity markers, are critical for tailoring management strategies. The progression to severe conditions like right ventricular failure, though uncommon, is a potential risk in advanced stages. It is crucial to identify specific biomarkers with a prognostic role in this category of patients. The question of when OSA patients should be measured for the occurrence of PH requires more data. Nevertheless, OSA patients with obesity and concomitant COPD seem to be an important subgroup in this context.

Treatment Impact: Treatment strategies, particularly CPAP and oxygen therapy, have shown promise in mitigating the progression of PH in OSA patients. However, the long-term efficacy of these treatments warrants further investigation. Similarly, the effect of other OSA treatment modalities (surgery, MAD) on the prognosis of concomitant PH is warranted. On the other hand, less is known regarding the effect of treatment for PH on the occurrence as well as the prognosis of OSA, which should stimulate future research directions.

## 7. Conclusions

The relationship between OSA and PH represents a fascinating intersection of respiratory and cardiovascular pathophysiology, with profound implications for patient care and clinical outcomes. This comprehensive review has delved into the intricacies of their pathophysiology, epidemiology, prognosis, and treatment, offering insights into their complex interplay and highlighting the challenges and opportunities in managing these conditions.

## Figures and Tables

**Table 1:** Summary of the articles addressing the association of obstructive Sleep apnea vs. pulmonary hypertension and the impact of CPAP treatment.

	OSA Criteria	Sample Size	Main Findings	Study Design	First Author	Year
Prevalence of PH among OSA patients	AHI ≥ 5	83 patients	Prevalence of PH in association with OSA is likely to be high	Cross-sectional	* Minai [16]	2009
	RDI > 20	100 Patients	PH is common in moderate and severe OSA	Observational	* Laks [18]	2009
	AHI > 20	220 patients	PH was observed in 17%	Observational	** Chaouat [19]	1996
	AHI > 5	92 patients	OSA can cause mild PH	Observational	** Sanner [20]	1997
	AHI > 5	44 patients	PH is frequent in patients with OSA	Cross-sectional	** Bady [21]	2000
Prevalence of OSA among PH patients	AHI > 5	71 patients	OSA is highly prevalent in patients with PH	Cross-sectional	* Spiesshoefer [22]	2021
	AHI > 5	140 Patients	OSA is common in PH	Observational	** Lu Yan [23]	2021
	AHI > 5	151 Patients	OSA observed in 38% of patients with PH	Observational	** Naguaka [24]	2018
Impact of PAP treatment on PH levels among OSA patients	AHI > 15	52 Patients	Improvement in the severity of PH	Observational	* Colish [26]	2012
	AHI > 20	65 Patients	Mean pulmonary artery pressure did not change	Observational	** Chaouat [28]	1997
	AHI > 15	20 Patients	Decrease in PH	Observational	* Sajkov [29]	2002
	AHI >15	33 Patients	Decrease in PH	RCT	* Arias [30]	2006
	AHI > 10	40 Patients	Decrease in PH	Case–control	* Duchna [31]	2006
	AHI >15	41 Patients	Decrease in PH	Case–control	* Alchanatis [32]	2001

Abbreviations: AHI = apnea hypopnea index; OSA = obstructive sleep apnea; PH = pulmonary artery hypertension; RCT = randomized controlled trial; RDI = respiratory desaturation index. * PAP measured by echocardiography; ** PAP measured by right heart catheterization.
